# Acetylation Mimics Within a Single Nucleosome Alter Local DNA Accessibility In Compacted Nucleosome Arrays

**DOI:** 10.1038/srep34808

**Published:** 2016-10-06

**Authors:** Laxmi N. Mishra, Sharon Pepenella, Ryan Rogge, Jeffrey C. Hansen, Jeffrey J. Hayes

**Affiliations:** 1 Department of Biochemistry & Biophysics, University of Rochester Medical Center, Rochester NY, 14642, USA; 2 Department of Biochemistry and Molecular Biology, Colorado State University, Fort Collins, CO, USA

## Abstract

The activation of a silent gene locus is thought to involve pioneering transcription factors that initiate changes in the local chromatin structure to increase promoter accessibility and binding of downstream effectors. To better understand the molecular requirements for the first steps of locus activation, we investigated whether acetylation of a single nucleosome is sufficient to alter DNA accessibility within a condensed 25-nucleosome array. We found that acetylation mimics within the histone H4 tail domain increased accessibility of the surrounding linker DNA, with the increased accessibility localized to the immediate vicinity of the modified nucleosome. In contrast, acetylation mimics within the H3 tail had little effect, but were able to synergize with H4 tail acetylation mimics to further increase accessibility. Moreover, replacement of the central nucleosome with a nucleosome free region also resulted in increased local, but not global DNA accessibility. Our results indicate that modification or disruption of only a single target nucleosome results in significant changes in local chromatin architecture and suggest that very localized chromatin modifications imparted by pioneer transcription factors are sufficient to initiate a cascade of events leading to promoter activation.

Chromatin is a dynamic nucleoprotein assemblage that exhibits local and global condensation *in vitro* and in the nucleus^1^,^2^. *In vitro*, extended 10 nm diameter “beads-on-a-string” structures fold into locally condensed structures such as the 30 nm diameter chromatin fiber^3^,^4^, and self-assemble into large oligomeric structures that are stabilized by interdigitated fiber-fiber interactions and appear to mimic the packaging of globally condensed chromatin in chromosomes^3^,^4^,^5^,^6^. The formation of folded and oligomeric chromatin structures is observed *in vitro* in the presence of divalent or multivalent cations with DNA templates assembled with only the core histone proteins^4^,^7^,^8^. Importantly in this regard, the disordered core histone N-terminal tail domains, which extend away from the structured nucleosome core, play an essential role in the assembly of locally and globally condensed chromatin structures^8^,^9^,^10^. Multiple distinct mechanisms are involved in tail domain mediated chromatin condensation. For local folding of the chromatin fiber, the H4 tail domain engages in protein-protein interactions with a H2A/H2B acidic patch on the surface of neighboring nucleosomes^11^,^12^,^13^. In contrast, all of the tail domains function in the assembly of chromatin oligomers^14^,^15^, acting at least in part through protein-DNA interactions^6^,^16^,^17^,^18^,^19^. Interestingly, crosslinking of the H4 tail at position 21 to a neighboring H2A/H2B acidic patch during 30 nm fiber formation traps the tail in its intra-array interactions and inhibits the ability of the array to form oligomers^20^. This demonstrates that folding and oligomerization are mechanistically distinct and highlights the importance of H4 tail rearrangement for assembly of condensed chromatin structures. Deciphering the molecular mechanisms of tail-mediated chromatin condensation is critical for understanding how the tail domains function biologically, particularly with respect to tail post-translational modifications such as acetylation.

Both the wrapping of DNA into nucleosomes and the formation of condensed chromatin structures restrict the accessibility of DNA to trans-acting factors^21^,^22^ and in general repress gene expression^23^. The transition from a repressive chromatin structure to an activated, transcriptionally permissible locus often involves the binding of ‘pioneer’ transcription factors that can associate with cognate sites either between or on the surface of nucleosomes and initiate a transition from a repressive chromatin structure to an activated chromatin locus^24^,^25^. Binding of such factors can directly alter nucleosomes or condensed chromatin structures^26^ or can recruit additional factors and activities to initiate chromatin remodeling or install activating posttranslational modifications on the core histone proteins^25^,^27^. Post-translational modifications of the tails (PTMs), including but not limited to acetylation, methylation and phosphorylation, are known to both hinder and enhance chromatin condensation and transcription^18^,^28^,^29^. For example, increased levels of acetylation of the histone H3 and H4 tail domains *in vivo* are associated with areas of open chromatin that is more accessible to DNA-binding factors, and promoters of transcriptionally active genes^30^,^31^,^32^. Acetylation of N-terminal tail domains in conjunction with gene activation can affect chromatin structure directly by altering histone tail interactions with both DNA and protein targets^33^,^34^ or indirectly by functioning as an epigenetic mark^35^. Moreover, *in vitro* studies have shown that acetylation of the core histones greatly reduces the propensity of oligonucleosome arrays to assemble into folded and oligomeric chromatin structures^36^,^37^,^38^.

Another localized change in chromatin structure that is associated with many active genes in fungi and metazoans is the so-called nucleosome-free region (NFR), in which canonical histone-DNA contacts are disrupted upstream of the start site of transcription, as well as downstream near the end of the gene^39^. NFRs are almost certainly depleted of core histone-DNA interactions due to the binding of general and specific transcription factors to promoter elements. Although one cannot rule out non-canonical histone-DNA interactions at NFRs, proteins such as SWR1 recognize NFRs in manner similar to that of naked DNA^40^,^41^. Thus, a NFR leads to a local discontinuity in the regularity of the chromatin fiber. In this regard, model studies show that a 12-mer nucleosome arrays containing a single histone-free gap in the array show reduced levels of chromatin fiber folding^42^. Therefore, a NFR may also lead to local decondensation of the chromatin fiber, thereby facilitating access of general and gene-specific transcription factors along with RNA Pol II at important gene regulatory sites.

Here, we engineer a novel *in vitro* model system to investigate whether histone H3 and H4 tail acetylation within a single nucleosome, or the presence of a NFR, is sufficient to enhance the local accessibility of DNA within condensed chromatin structures *in vitro*. Specifically, we insert a unique nucleosome in the center of a 25-mer nucleosome array and quantify local and global linker DNA accessibility of the chromatin at different salt concentrations using restriction enzyme accessibility assays. Our results indicate that mimetics of acetylated lysine residues in the H4 tail domains of the central nucleosome causes a significant increase in local DNA accessibility within condensed chromatin structures. Likewise, we find that a NFR greatly increases local but not global accessibility within the condensed array. These results suggest that histone acetylation and NFRs function in part by locally disrupting the condensed state of the chromatin fiber, thereby increasing the accessibility of the chromatin to protein factors in specific regions of the genome.

## Results

**Figure 1:**
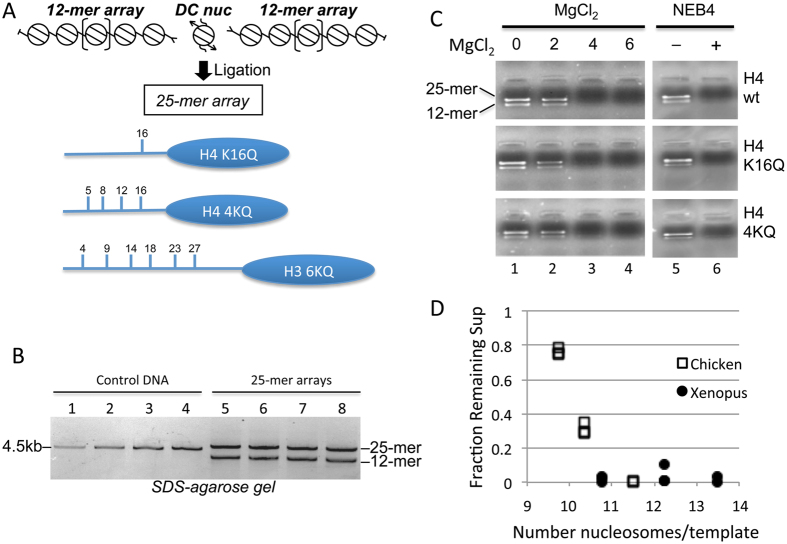
Histones and nucleosome arrays. (**A**) Positions of lysine acetylation mimics and ligation strategy. *Top*, Schematic showing arrangement of 12-mer arrays and DC nucleosome upon ligation to the 25-mer nucleosome array. Directional ligation sites are indicted by arrowheads, t-bars indicate unligatable DNA ends. *Bottom*. The locations of lysine to glutamine substitutions within H3 and H4 used in this study are shown. (**B**) Increasing amounts of the 4.5 kb control DNA (1, 2, 3, and 4 μg) are loaded in lanes 1–4. Ligated WT, H4 4KQ, H3 6KQ and H3 6KQ/H4 4KQ arrays are shown (lanes 5–8). Some unligated 12-mer array remains after ligation. (**C**) Ligated arrays are saturated with nucleosomes and completely self-associated in NEB4 digestion buffer. Self-association assays with the arrays (right) were performed as described with increasing MgCl^2+^ (lanes 1–4), as indicated. Self-association of arrays in the absence (−) or presence (+) of NEB4 buffer. (**D**) Self-association and analytical ultracentrifugation (AU) analysis of arrays in NEB4 buffer. 12-mer nucleosome array templates were reconstituted to increasing saturations with either chicken or recombinant *Xenopus* core histones and the level of saturation determined by AU. The fraction of each array remaining in solution as determined by self-association assays was plotted vs number of nucleosomes per template. Duplicates of assays are shown, 12 nucleosomes per template is considered saturation.

In native chromatin, nucleosome-nucleosome interactions and formation of higher order structures restrict accessibility of cognate sites important for gene expression. We wished to determine whether modification of a single nucleosome in the midst of an otherwise unmodified nucleosome array is sufficient to increase local and/or global DNA accessibility of proteins to their cognate DNA binding sites in chromatin. We exploited acetylation mimetics within the H3 and H4 N-terminal tail domains as acetylation is known to be well correlated with gene activation^31^ and to have direct impacts on chromatin compaction^36^,^38^,^43^,^44^. We designed a system whereby a single recombinant nucleosome (DC nucleosome) was ligated between two independently reconstituted 12-mer nucleosome arrays to form a 25-nucleosome array (Fig. 1A). Ligation was facilitated by the directional overhangs created by DraIII digestion, which do not allow the single DC nucleosome or the 12-mer array to ligate to themselves.

DC nucleosomes containing specific lysine → glutamine (K → Q) substitutions, as mimics of acetylated lysine within the H3 and H4 tail domains, were prepared as described in the Materials and Methods. K → Q substitutions within the H4 tail domain previously have been shown to recapitulate the effect of acetylation with regard to array folding and self-association^17^,^37^. DC nucleosomes were assembled from combinations of native core histones and H4 and H3 containing single (H4 K16Q) or multiple (H4 4KQ, H3 6KQ) K → Q substitutions as outlined in Fig. 1A. The DC nucleosomes were gradient-purified and ligated with 12-mer arrays reconstituted with native core histones to create 25-mer oligonucleosome arrays with the DC nucleosome at the center of the array (Fig. 1B). The native 12-mer arrays were employed in excess to maximize incorporation of the DC nucleosomes into the 25-mer arrays. 25-mer arrays (Two 12-mer arrays ligated to central DC nucleosome) were easily distinguishable from ligated 13-mer arrays (only one 12-mer ligated to DC nucleosome) based on their mobility in SDS-agarose gels. To ensure these 25-mer arrays remained fully saturated with nucleosomes after ligation and buffer exchange, arrays were analyzed by self-association assays, which are sensitive to the degree of arrays saturation^15^,^45^. Results indicated that the three ligated 25-mer arrays consistently exhibited self-association in the presence of increasing concentrations of MgCl_2_ that was equivalent to that of the saturated 12-mer arrays and consistent with nucleosome saturation (Fig. 1C, lanes 1–4).

In order to assess the effect of acetylation mimics, we determined the accessibility of the DraIII sites bracketing the DC nucleosome in the ligated 25-mer arrays by following the kinetics of digestion by this restriction enzyme. Of note, binding and cleavage of restriction enzymes is affected by nucleosome structure in a manner identical to that of the activity of sequence-specific DNA binding transcription factors^46^. Digestions were done in buffer specified by the manufacturer, which contains 10 mM magnesium acetate and 50 mM potassium acetate (see Methods). This concentration of magnesium ions is sufficient to induce complete self-association of saturated arrays. Indeed, differential centrifugation studies of 12-mer arrays assembled with either histones isolated from chicken erythrocytes or recombinant *Xenopus* core histones show that arrays fully saturated with nucleosomes are completely self-associated in these buffer conditions (Fig. 1D). Moreover, the differential centrifugation assays further show that nearly all of the ligated 25-mer arrays self-associate in the DraIII digestion buffer (Fig. 1C, lane 6). Recent studies have shown that the self-associated oligomers formed under these ionic conditions are chromosome-sized and stabilized by interdigitated interaction of extended 10 nm fibers^6^. Thus, experiments performed under standard digestion buffer conditions will measure DraIII accessibility to linker DNA within globally packaged nucleosome arrays similar to that found in native chromosomes^6^.

**Figure 2:**
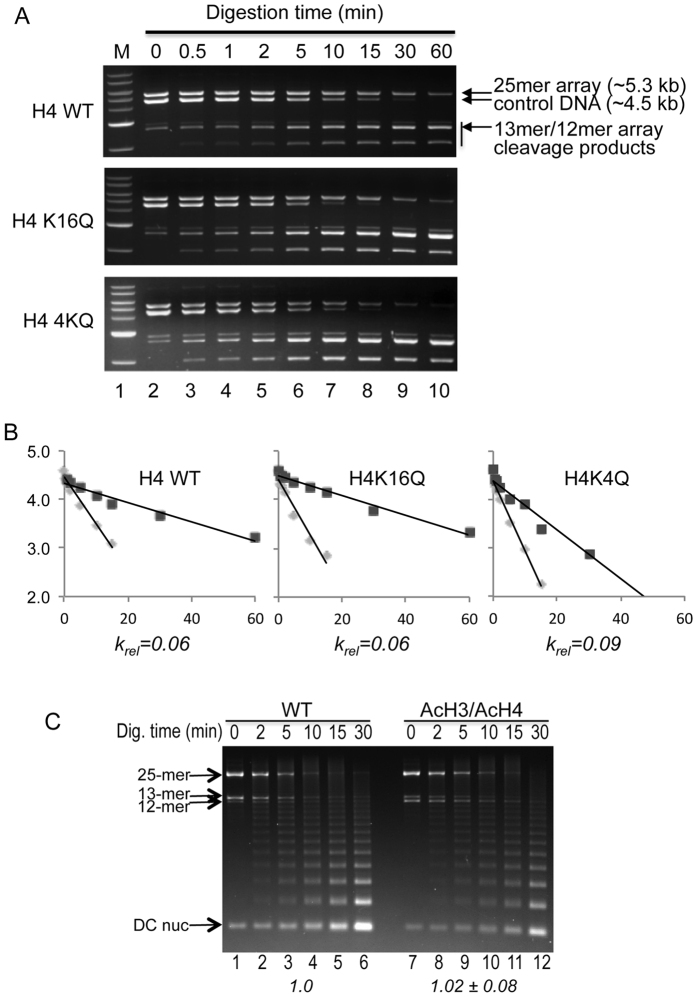
Accessibility of the central nucleosome linker DNA within a 25-nucleosome array. (**A**) Example of digestion time course for arrays containing wt H4, H4 K16Q or H4 4KQ within the central DC nucleosome. Time points (min.) of digestion are shown at the top of the gel. Digestion products were analyzed on 0.7% SDS-agarose gels followed by EtBr staining. Bands corresponding to the 5.3 kb 25-mer template, the 4.5 kb naked DNA control, and residual 12/13 mer arrays and other digestion products are indicated. Lane 1 contains molecular size markers (M). (**B**) Example plots of quantified digestion data as described in Materials and Methods. Lines represent linear regression fits with R^2^ for each fit ranging from 0.96 to 0.98. The rate of the chromatin digestion relative to the naked DNA internal control (k_rel_) is indicated below each plot. (**C**) Effects of acetylation mimics are localized to the DC nucleosome in the 25-mer arrays. The 25-mer arrays were digested with Eco RI and products run on an SDS-agarose gel. Note that EcoRI sites lie between each nucleosome in the 25-mer array. Relative rate of the loss of the uncut 25-mer normalized to WT is indicated below the gel.

In control experiments we examined DraIII accessibility of naked DNA compared to chromatinized templates. To allow for precise normalization of restriction enzyme digestion activity between reactions, a naked 4.5 kb DNA fragment containing a single DraIII site was added to each 25-mer nucleosome array sample (Fig. 2A). The naked DNA fragment runs just below the nucleosome array DNA template in SDS-agarose gels and was carefully matched to the concentration of the chromatin template (Figs 1B and 2A). Digestion was initiated by the addition of appropriate amounts of DraIII and products analyzed on SDS-agarose gels. We observed that both naked DNA and oligomeric chromatin structures were cut by the enzyme, with the naked DNA digesting at a faster rate than the nucleosome arrays. Quantification of the products showed that cleavage of the full-length templates occurred with apparent first-order kinetics and showed excellent fits to single exponential decay curves that, importantly, included a majority of the substrate in each reaction (Fig. 2B). Comparison of the absolute rates of cleavage of the chromatin and naked templates (see Methods) allowed calculation of a relative rate of cleavage (k_rel_) that corrects for small differences in restriction enzyme activity between samples. Typically we observed a ~20-fold difference in the absolute rate of cleavage between the naked DNA and nucleosome arrays, indicating that packaging of the arrays into chromatin oligomers resulted in a significant reduction in accessibility of the DC nucleosome linker DNA. Nevertheless, the bulk of 25-mer arrays were efficiently digested by DraIII, despite being packaged into chromosome-sized oligomers. Moreover, we find k_rel_ was not dependent on enzyme concentration (results not shown). Together these findings suggest that the oligomeric chromatin structure provides a thermodynamic but not a kinetic impediment to linker DNA accessibility, and thus serves as a good model for the concentration-dependent DNA binding activity of trans-acting factors in packaged chromatin (see Discussion).

We next determined whether installation of mimics of acetylated lysine at positions 5, 8, 12, and 16 within the H4 tail domain of the DC nucleosome had any effect on the accessibility of the DC nucleosome linker DNA. Interestingly, inclusion of H4 4KQ in the DC nucleosome resulted in an increase of about 50% in the overall rate of digestion of the linker DNA abutting this nucleosome, compared to arrays containing wt H4 (Fig. 2B; Table 1). This result implies that tetra-acetylation of a single set of H4 tail domains within a single nucleosome can disrupt the local packaging of nucleosome arrays within the chromatin oligomers. To determine if a single nucleosome containing the H4 K16Q modification was able to increase accessibility of linker DNA, we determined relative rates of DraIII digestion of 25-mer arrays containing H4 K16Q in the DC nucleosome. Comparison to the relative rate of digestion of control arrays indicates that this single modification alone was unable to cause a detectable increase in the accessibility of the DC nucleosome linker DNA (Fig. 2B, Table 1).

Acetylation has been reported for at least six residues within the H3 tail domain, at lysines 4, 9, 14, 18, 23, and 27. To investigate potential effects of acetylation within the H3 tail domain, we generated an H3 in which all six of these residues were swapped for glutamine (H3 6KQ, Fig. 1A). Somewhat surprisingly, incorporation of H3 6KQ within the DC nucleosome did not result in a detectable increase in linker DNA accessibility compared to the unmodified control in our assay (Table 1). However, we found that including both H3 6KQ and H4 4KQ in the DC nucleosome resulted in an additional increase in accessibility over that observed with H4 4KQ alone indicating that H3 6KQ was able to synergize with H4 acetylation mimics to increase accessibility of the linker DNA adjacent to the DC nucleosome (Table 1).

We next asked whether the effects of the acetylation were only localized to the vicinity of the DC nucleosome, or extended more broadly throughout the 25-nucleosome array. To do so, we assessed the rate of cleavage of EcoRI restriction sites, which are located between all nucleosomes within 25-mer arrays, for arrays containing either wt H4 or H3 6KQ/H4 4KQ within the DC nucleosome. We found no detectable difference in the rate of cleavage by EcoRI between these arrays, when measured either by loss of the full-length template or accumulation of small oligosomes (Fig. 2C, and results not shown). This result indicates that while the acetylation mimics within the DC nucleosome alter local accessibility of the adjacent linker DNA, they do not cause detectable changes in the bulk accessibility of linker DNA segments throughout the packaged arrays.

**Figure 3:**
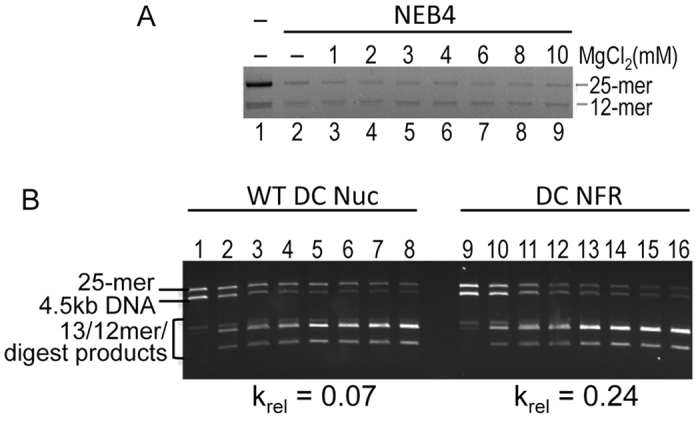
Effect of a nucleosome free region on DC Linker DNA accessibility. (**A**) A 25-mer array with a nucleosome-free region is maximally self-associated in DraIII digestion buffer. Lanes 1 and 2 show arrays soluble in the absence and presence of NEB4 buffer. Lanes 3–9 show soluble arrays in NEB 4 buffer with increasing concentrations of additional MgCl_2_, as indicated. (**B**) A Nucleosome free region enhances accessibility of DraIII sites in the center of the 25-nucleosome array. DraIII digestions were performed with 25-mer arrays in which the central nucleosome contained native histones (WT, lanes 1–8) or was ligated in as a naked template representing a nucleosome-free region (DC NFR, lanes 9–16). Digestions were as described for 0, 2, 4, 6, 8, 10, 15, 30, 45, and 60 minutes. k_rel_ calculated for each of the digests is shown below the gels.

Promoters of many active genes in yeast and higher organisms contain a ‘nucleosome free region’ (NFR) immediately upstream of the transcription start site in which detection of histone-DNA interactions is reduced^47^. To determine the extent to which a NFR increases local DNA accessibility within a large nucleosome array, we replaced the DC nucleosome with the naked DC template and repeated the experiment. Self-association experiments show that arrays with a NFR region are nearly fully assembled into large oligomers in the DraIII digestion buffer, similar to the extent of self-association induced by high levels of MgCl_2_ (Fig. 3A). Nevertheless, we find that DraIII sites in the NFR in the center of the 25-nucleosome array are cleaved at rates ~4-fold faster compared to control arrays in which the DC nucleosome template is assembled with unmodified core histones, and only about 4-fold less than that of the naked DNA (Fig. 3B). In contrast, EcoRI digestions indicate that the rest of the NFR-arrays remain as inaccessible to this enzyme as control arrays in which unmodified core histones are assembled on the DC nucleosome template (results not shown). These results suggest that the NFR creates a distinct structural discontinuity in the center of a packaged oligomeric nucleosome array.

**Figure 4:**
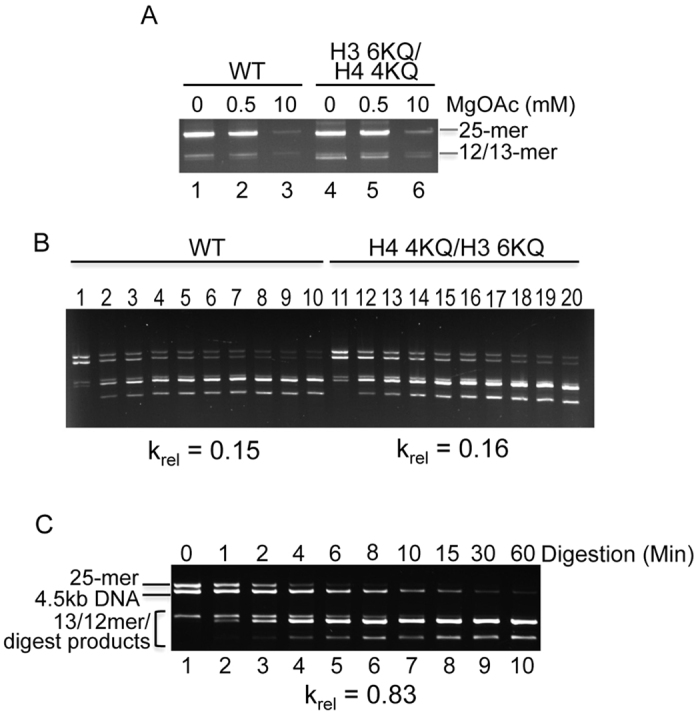
Effect of reduced chromatin compaction on DC Linker DNA accessibility. 25-mer Arrays were assembled with either unmodified histones (WT) or H3 6KQ/H4 4KQ in the central DC nucleosome. (**A**) Arrays are not self-associated in digestion buffer containing 0.5 mM magnesium acetate. DraIII digestion buffer was prepared containing either 0.5 mM or 10 mM magnesium acetate (lanes 2 and 5 or 3 and 6, respectively) and the extent of self-association determined by a centrifuge assay. Arrays in TE buffer are shown for comparison (lanes 1 and 4). (**B**) Effect of reduced self-association on DC nucleosome linker DNA accessibility. The relative rate (krel) of DraIII digestion in buffer containing 0.5 mM magnesium acetate was determined. krels are indicated below the gels. (**C**) DC nucleosomes were ligated with naked p12 templates and the relative rate of digestion in NEB4 buffer determined. Lanes 1–10 show digestion time points taken over 60 minutes, as indicated.

In our assays, the specific ionic conditions found in the DraIII digestion buffer cause self-association of the arrays into higher order oligomeric chromatin structures that are packaged as 10 nm fibers^6^. At lower salt concentrations, individual nucleosome arrays fold rather than self-associate. However in both low-Mg (folded) and high Mg (self-associated) states involve direct nucleosome-nucleosome interactions. To document effects of acetylation on folding, we reduced the [Mg^2+^] to 0.5 mM in the digestion buffer. Under these conditions self-association is expected to be eliminated, and the individual nucleosome arrays fold into compacted fibers^6^. Indeed, differential centrifugation assays show that the arrays remain completely unassociated in the low Mg^2+^ buffer (Fig. 4A). Interestingly, DraIII digestion assays revealed that arrays containing unmodified (WT) histones in the DC nucleosome were cleaved with a k_rel_ (0.15 ± 0.02) only ~2-fold faster than that observed with the same arrays when self-associated in the high-magnesium buffer (Fig. 4B, Table 1). Moreover, arrays with H3 6KQ/H4 4KQ in the DC nucleosome are digested at approximately equivalent rates in the low and high Mg^2+^ buffers (Table 1). To ascertain whether the remaining reduction in digestion rate relative to free DNA derives from local folding of the nucleosomes arrays, or is due to direct steric interference by the DC nucleosome on linker DNA accessibility^48^, we ligated DC nucleosomes to naked 12-mer array templates and examined the DraIII digestion rate. Interestingly, such arrays digested at rates nearly equivalent that of the naked DNA control (Fig. 4C and Table 1), indicating that the mere presence of a nucleosome on the DC template does not significantly reduce DraIII accessibility throughout the array. These results suggests that nucleosome-nucleosome interactions, found in both low- and high-Mg^2+^ states, appear to be primarily responsible for the substantial reduction in linker DNA accessibility in condensed chromatin (see Discussion).

## Discussion

In this work we provide evidence that acetylation of a single histone tail domain within a single nucleosome is sufficient to alter accessibility of the adjoining linker DNA within condensed chromatin. Such a situation is likely to occur during the earliest stages of activation of a silent locus upon binding of a ‘pioneer’ transcription factor. Pioneer factors have been shown to be able to invade silent chromatin and bind to either linker DNA or to DNA on the nucleosome surface^25^. In our system, we choose to measure accessibility by assessing the digestion rate of the restriction enzyme DraIII. This enzyme (25.7 kDa) serves well as a model for pioneer transcription factors which have similar molecular weights^49^,^50^. The linker DNA in our 25-nucleosome array is about 15-fold less accessible than the unchromatinized naked DNA template. Acetylation mimics within the H4 tail domain of the central DC nucleosome resulted in a ~50% increase in accessibility of the adjacent linker DNA, and while acetylation mimics within the H3 tail did not have a significant effect on their own, the modified H3 tail was able to synergize with the H4 acetylation mimics to further increase accessibility. This increase in accessibility appeared localized to the immediate vicinity of the modified DC nucleosome as the acetylation mimics had no effect on EcoRI digestion within the linker DNAs in the rest of the array, or on the ability of the arrays to undergo Mg^2+^-dependent self-association.

The observation that H4 acetylation mimics within a single nucleosome disrupt local chromatin structure enough to allow greater access to the DC linker DNA is somewhat surprising, as the sheer size of the 25-nucleosome array would suggest that modification of a single set of tail interactions would have a relatively minor effect. While acetylated regions in the vicinity of active promoters typically span several nucleosomes, our data indicate that a pioneering acetylation event on a single nucleosome can alter chromatin structure sufficient to lead to more robust and wide-spread modification^51^. Indeed, localized disruption of structure may contribute to ‘spreading’ of acetylated regions in a manner that does not require direct physical interactions of factors with an initially acetylated nucleosome, as the most accessible nucleosomes for subsequent HAT activity would be adjacent to the primary acetylated nucleosomes. It will be interesting in future experiments to investigate the extent to which acetylation of increasing numbers of adjacent nucleosomes further increases DNA accessibility within the condensed chromatin.

Given the rather modest effects of acetylation on the structure and accessibility of DNA within individual nucleosomes^52^,^53^,^54^,^55^, and the much greater impact of acetylation on the stability of higher order chromatin structure^36^,^56^, it is highly likely that the effects of acetylation mimics we document involve localized disruption of nucleosome-nucleosome interactions that stabilize higher-order chromatin structures. Importantly, linker DNA accessibility is reduced ~13-fold in 10 mM Mg^2+^ and ~7-fold in 0.5 mM Mg^2+^ buffer compared to naked DNA, indicating that significant impediment to linker DNA accessibilty persists in low Mg buffer lacking array self-association. It is interesting to note that both low and high Mg^2+^ states involve nucleosome-nucleosome interactions, however recent data indicates that these interactions are derived from fundamentally different secondary structurs in each state. Arrays in low (0.5 mM) Mg^2+^ have been shown to adopt folded chromatin fiber states featuring local (intra-array) nucleosome-nucleosome interactions, while arrays in high Mg^2+^ form large self-associated structures in which nucleosomes participate in inter-array interactions with interdigitated inter-array nucleosome-nucleosome interactions between 10 nm filaments^6^,^17^,^18^. Thus, our data indicate that either intra- or inter-array nucleosome-nucleosome interactions are of primary consequence in restriction of DNA accessibility in chromatin. Importantly, we observe that while installation of acetylation mimics increases linker DNA accessbility in high Mg^2+^ conditions, there is little effect of these modifications on arrays in low-Mg^2+^ conditions (Table 1). This suggests that inter-digitated nucleosome-nucleosome interactions between 10 nm filaments in high Mg^2+^ may be more susceptable to disruption than those involved in the formation of folded chromatin fibers.

Experiments with a single, naked nucleosome template modeling an NFR in the middle of the 25-mer array support this conclusion. We find that cleavage of the DraIII sites within the NFR occurs at a rate about 4-fold less than that of the naked DNA control while EcoRI cleavage and self-association experiments indicate that rest of the chromatin remains as highly condensed as arrays with WT histones in the DC nucleosome. Thus, the acetylation mimics in the DC nucleosome, and the NFR result in a localized discontinuity in the condensed structure due to disruption of nucleosome-nucleosome interactions. The lack of effect on accessibility of the bulk of the linker DNA segments within the condensed arrays suggests the disruption may require a specific arrangement exposing the DC nucleosome on the surface of the otherwise condensed chromatin structure, or exposure may be facilitated by the dynamic nature of the chromatin^22^,^26^,^57^. This would be consistent with the previously characterized rapid pre-equilibrium mechanism of site exposure for DNA sequences within the nucleosome core, and suggests the overall condensed chromatin fiber rapidly equilibrates with states in which the linker DNA is accessible to DNA binding factors. Thus, the fold-reduction (k_rel_) we determine would then equate to the probability of a local excursion to an exposed conformation from the condensed, inaccessible state.

H3 acetylation, as well as H4 acetylation, is highly correlated with promoters of actively transcribed genes^31^. However, we found that six K → Q substitutions within the H3 tail domain did not significantly increase accessibility within the 25-nucleosome array, suggesting that acetylation of this tail domain is largely involved in nucleosome-specific signaling to chromatin ‘readers’^29^. Consistent with this idea, we previously found that installation of acetylation mimics in the H3 tail domain have a surprisingly modest effect on the ability of arrays to undergo Mg^2+^-dependent self-association^37^. However, H3 6KQ synergized with H4 4KQ to further increase accessibility of the DC linker DNA. It is interesting to note that although H3 6KQ caused only a modest reduction in Mg-dependent self-association, a much larger increase was observed when both the H3 and H4 acetylation mimics were within the same array^37^. Thus, in addition to directing binding of ancillary factors to indirectly alter chromatin structure, H3 acetylation may also be ‘designed’ to augment the direct effect of H4 acetylation.

Previous work suggests that H4K16 acetylation might be a key event in disrupting intra-array interactions and condensation. H4K16 is thought to participate in protein-protein interactions with the H2A/H2B acidic patch of neighboring nucleosomes^11^. Acetylation of H4K16 abrogates this interaction in both long and short fibers^33^,^38^,^43^ and reduces the capacity to oligomerize^33^. However, these acetylation experiments were performed utilizing arrays in which all nucleosomes contained the acetylated residue on the H4 tail. Our results indicate that H4K16Q within a single nucleosome is not sufficient to disrupt folding, but the addition of 3 more lysine mimics to the H4 tail increases access to DNA by 50%. Previous evidence demonstrates that glutamine substitutions and lysine acetylation within the H4 tail have identical effects on core histone tail binding within nucleosome cores^55^ and nucleosome array self-association^37^. However, recent studies argue that H4K16 acetylation, but not the H4K16 lysine to glutamine substitution, drastically affects chromatin folding^38^, suggesting the possibility that actual acetylation of H4K16 may cause a local disruption. Notably, this same study demonstrates that both acetylation and acetylation mimics of all 4 lysines on the H4 tail within an array disrupt folding to a greater extent than H4K16-acetylation or H4K16Q substitution. However, acetylation or acetylation mimics of lysines 5, 8 and 12 showed little disruption of folding. This demonstrates that either the additive effect of at least 4 acetylation events per tail, or H4K16 acetylation working in concert with acetylation at the other 3 sites, is important for chromatin structure disruption, and is consistent with our observation. Thus, our results suggest that acetylation of all four lysines within the H4 tail of a single nucleosome may be required to increase DNA access and initiate locus activation. Further, although the effects we measure are only about 2-fold, we note that other biological processes (e.g. dosage compensation) have similar magnitudes and result in critical biological outcomes. Moreover, although we did not investigate the role of linker histones in DNA accessibility, it is possible that H1 would accentuate the effects of single-nucleosome acetylation described here, as acetylation is known to weaken H1-mediation of inter-nucleosome interactions within chromatin and to override H1’s stabilizing influence on inter-nucleosome interactions of the H3 and H4 tail domains^17^,^58^,^59^.

It has been suggested that in addition to recruitment of histone modifying activities such as histone acetyl transferases, physical binding of pioneer transcription factors may alter nucleosome or chromatin structure directly to initiate events leading to activation of the locus^25^. Indeed, the non-specific binding of a bacterial transcription factor LexA induces decondensation of a trinucleosome, likely by binding to the linker DNA when present at very high concentrations^26^. Thus, it will be interesting to use the model system presented herein to insert cognate sites for known pioneer factors in both the DC nucleosome core and linker DNA to test the effect of binding on local DNA accessibility. Interestingly, we find that accessibility of the DC nucleosome linker DNA is reduced by ~15 fold as a result of chromatin compaction, consistent with previous investigations^22^,^26^. This is important because only about 75% of DNA in chromatin is directly occluded by tight association with the core histone proteins. Our results predict that binding activities for factors targeted to the linker DNA would be reduced by a commensurate amount. However, careful measurements of binding affinity may reveal that certain pioneer factors have a unique ability to compensate for the expected impediments of folded chromatin.

## Materials and Methods

### Expression and purification of core histone proteins

Wild type and mutant core histones were expressed and purified as described previously^37^,^60^. Briefly, coding sequences for *Xenopus* histones H3 and H4 containing specific lysine-to-glutamine substitutions were obtained using the Stratagene QuikChange site-directed mutagenesis kit. *E. coli* BL21(DE3)pLysS cells were transformed with the pET expression plasmids harboring the expression vectors and were grown in 100 ml LB containing ampicillin (50 μg/ml) and chloramphenicol (34 μg/ml) until the OD_600_ reached 0.6. Expression of histones was induced by 0.4 mM IPTG for 3 h at 37 °C. Cells were harvested by centrifugation at 6000 × g for 10 min and pellets resuspended in 10 ml buffer containing 25 mM Tris pH 8.0, 5 mM EDTA. After resuspension, 100 μl of 100 mM PMSF, 200 μl of 10% Triton X- 100, 10 μl of 1M DTT, and 50 μl of 50 mg/ml lysozyme was added and the lysate was incubated at room temperature for 1 h, followed by incubation on ice for 10 min. Lysates were sonicated (Branson Sonifier, 30% duty cycle, output 4 for 10 seconds) on ice, and spun down for 30 min at 20,000× g. H3/H4 tetramers were prepared from the disrupted cells as described^60^. Histone H2A/H2B dimer was expressed and purified as described^61^.

### Preparation of DNA templates

A 204 bp DNA fragment containing the 601 nucleosome positioning sequence and asymmetric DraIII overhangs on both ends (DC template) was prepared as described previously^60^ with minor modifications. Briefly, the DC DNA fragment was PCR amplified using primers TCCCTCGGGCACGAGGTGGATATCGGACCCTATACGCGGC and TCCCTCGGGCACGAGGTGAGTATTAATTAATATGAATTCGG, adding asymmetric AvaI sites to each end. The fragments were digested with AvaI and ligated, generating a fragment containing 4 tandem copies of the DC template with spacers. HindIII and BamH1 sites were added to the fragment PCR amplification with primers GCGAAGCTTCACGAGGTGGATATCGGACCCTATACGCGGC and GCGGGATCCCACGAGGTGAGTATTAATTAATATGAATTCGG, and the resulting fragment was digested and ligated into a pBS(II)SK(+) vector. The plasmid was transformed into DH5α cells, and isolated using a giga prep kit (Qiagen). Ten mg of the purified plasmid was digested with DraIII HF enzyme (New England Biolabs, NEB), followed by gel isolation, ethanol precipitation, and resuspension of the 204 bp DC DNA fragment into 500 μl TE.

A 2.5 kb DNA fragment containing twelve tandem repeats of a *Lytechinus variegates* 5S rDNA was prepared from the p12-5S-C1 plasmid as described previously^59^. The final 12-mer DNA fragment excised from the plasmid contained a complimentary asymmetric DraIII overhang to the DC DNA plus a non-ligateable dephosphorylated end. A ~4.5 kb DNA fragment was derived from the non-array portion of the p12-5S-C1 plasmid as an internal control for the 25-mer array linker DNA accessibility assays. Typically, 60 μg of this p12 control DNA was prepared from 100 μg p12-5S-C1 plasmid, incubated at 37 °C overnight with 10 μl SalI (200 units) and 8 μl AlwN1 (80 units), in 1X NEB3 buffer and BSA (300 ng/μl) in a final volume of 300 μl. The resulting p12 control DNA fragment was gel isolated by electroelution, ethanol precipitated, resuspended in 200 μl TE and stored at −20 °C.

### *In vitro* reconstitution of nucleosomes and nucleosome arrays

Reconstitution and purification of DC mononucleosomes and 12-mer nucleosome arrays was carried out as described previously^37^,^60^, with recombinant *Xenopus* core histones and core histones purified from chicken erythrocytes, respectively. Ligation of DC mononucleosomes was carried out with excess 12-mer arrays to ensure complete assembly of the monosomes into 25-mer arrays. The ligation reactions typically contained 20 ng sucrose gradient-purified DC nucleosomes, 500 ng 12-mer arrays, and 1 μl (400 units) T4 DNA ligase, in ligation buffer (0.2 μg BSA, 1 mM ATP, 10 mM MgCl_2_, 0.25 mM EDTA, and 10 mM Tris, pH 8.0) in a final volume of 50 μl. The 25-mer arrays were subjected to buffer exchange through microfiltration^60^. All array preparations were checked for nucleosome saturation by self-association assays^15^.

### Quantification of 25-mer array DNA and identification of krel rates of cleavage

Analytical DraIII digestions were performed with 25-mer arrays in which the central DC nucleosome contained either native histones (WT), H4 K5Q K8Q K12Q K16Q (H4 4KQ), H4 K16Q (H4 K16Q), H3 K4Q K9Q K14Q K18Q K23Q K27Q (H3 6KQ), or H4 4KQ/H3 6KQ. Twenty μl of each array preparation (~1 μg) was mixed with an equal amount of p12 DNA control, in 110 μl final volume containing 1X NEB 4 digestion buffer (50 mM potassium acetate, 20 mM tris-acetate, 10 mM magnesium acetate, 1 mM DTT, pH 7.9). The amount of p12 control DNA was carefully matched to the chromatin by comparison to the ligated 25-mer product on 0.8% SDS-agarose gels (Fig. 1B). Ten μl of the solution was removed as an undigested control (t = 0 min) and added to 2 μl 6x SDS-native gel loading solution (10 mM Tris-HCl, pH 8.0, 1 mM EDTA, 30% glycerol, 1% SDS, 0.25% bromophenol blue dye). The remaining 100 μl was warmed to 37 °C and 2 μl of DraIII HF enzyme (diluted 1:5 to 4 U/μl) was added, followed by rapid mixing and further incubation at 37 °C. Ten μl samples were removed at time points as indicated in the figures and legends, and immediately mixed with 2 μl 6x SDS-native gel loading solution. Samples were run on a 0.8% SDS-agarose gels, the gels imaged on a BioRad Imager (Gel Doc^TM^ XR+), and the 25-mer array DNA bands and p12 control DNA bands quantified with the volume tools in Image Lab. The rates of digestion were determined by plotting ln(fraction uncut) vs time for both array and naked DNA bands and the data fitted to a simple y = mx + b equation in Microsoft Excel. Typically the first time point was omitted from the fits for the chromatin data due to obvious non-linearity of these points. This was due to a small fraction of the sample (5–15%) that digested with kinetics similar to that of the free DNA. Likewise, the last 2–4 time points were omitted from the naked DNA fits, as digestion of the last few % of the naked DNA occurred with much slower apparent kinetics. To allow comparison of the absolute rates of cleavage for chromatin and DNA templates within each sample, we also determined the relative rates of cleavage for the two naked DNA templates (results not shown). We found that the naked 25-mer template, containing two DraIII sites, was cleaved 4.2 ± 0.4-fold faster than the 4.5 kb control template, which contains a single DraIII site, indicating that there is an inherent difference in the overall probability of a single cleavage event of about 2-fold between the two DNAs. A parameter k_rel_ (k relative) rates was thus calculated as (slope chromatin/4.2x slope DNA) for each of the 25-mer arrays, correcting for the differences in inherent rates between the two templates.

## Additional Information

**How to cite this article**: Mishra, L. N. *et al*. Acetylation Mimics Within a Single Nucleosome Alter Local DNA Accessibility In Compacted Nucleosome Arrays. *Sci. Rep*. **6**, 34808; doi: 10.1038/srep34808 (2016).

## Figures and Tables

**Table 1:** Table of accessibilities of DC linker DNA relative to control DNA (k_rel_) in arrays containing the indicated histones assembled on the DC template or the naked DC template representing a nucleosome-free region.

DC Nucleosome	k_rel_		k_rel_/WT	
	10 mM Mg	0.5 mM Mg	10 mM Mg	0.5 mM Mg
WT	0.08 ± 0.01	0.15 ± 0.02	1	1.95 ± 0.18
H4 4KQ	0.11 ± 0.01		1.49 ± 0.12	
H3 6KQ	0.08 ± 0.01		1.00 ± 0.15	
H3 6KQ/H4 4KQ	0.14 ± 0.01	0.160 ± 0.01	1.88 ± 0.16	2.11 ± 0.17
Naked (NFR)	0.26 ± 0.02		3.62 ± 0.09	
p12-WT-p12	0.83 ± 0.05		10.9 ± 0.16	

The construct containing a single nucleosome flanked by two naked p12 DNA templates is indicated by p12-WT-p12. Accessibilities normalized to arrays in which the DC nucleosome contains unmodified histones are shown (k_rel_/WT).
